# The efficacy of Ahmed glaucoma valve drainage devices in cases of adult refractory glaucoma in Indian eyes

**DOI:** 10.4103/0301-4738.55068

**Published:** 2009

**Authors:** Jitendra K S Parihar, Devendra P Vats, Rakesh Maggon, Vijay Mathur, Anirudh Singh, Sanjay K Mishra

**Affiliations:** Department of Ophthalmology, Army Hospital (Research and Referral) Cantt -10, Delhi, India

**Keywords:** Ahmed glaucoma valve, adult refractory glaucoma, post traumatic glaucoma, complications

## Abstract

**Aim:**

To evaluate the efficacy of Ahmed glaucoma valve (AGV) drainage devices in cases of adult refractory glaucoma in Indian eyes.

**Settings and Design:**

Retrospective interventional case series study.

**Materials and Methods:**

Fifty two eyes of 32 patients of refractory glaucoma in the age group of 35 to 60 years who underwent AGV implantation with or without concomitant procedures from January 2003 to Jan 2007 were studied. Of these, 46 eyes (88%) had undergone filtering surgery earlier whereas remaining eyes underwent primary AGV implantation following failure of maximal medical therapy. The follow up ranged between 12 months to 48 months

**Results:**

Eighteen eyes (35%) had undergone phacoemulsification with AGV implantation, penetrating keratoplasty (PK) with AGV and intraocular lens (IOL) implantation in 13 eyes (25%), AGV over preexisting IOL in eight eyes (15%). AGV implantation alone was done in six (11%) eyes. Anterior chamber (AC) reconstruction with secondary IOL and AGV was performed in the remaining eyes. The mean intra ocular pressure (IOP) decreased from 36.3 ± 15.7 mm Hg to 19.6 ± 9.2 mm Hg. Complete success as per criteria was achieved in 46 eyes (88%). None of the eyes had failure to maintain IOP control following AGV.

**Conclusion:**

The AGV resulted in effective and sustained control of IOP in cases of adult refractory glaucoma in intermediate follow up.

The term refractory glaucoma is being used for any kind of glaucoma which has not responded to medical or surgical treatment and needs subsequent surgical re-intervention.[1,2] Management of adult refractory glaucoma is a challenging task for any glaucoma surgeon. The peculiarities involved with deranged anatomical configuration, physiology and dynamics of aqueous circulation is far different from other glaucoma patients.

The long-term follow-up of such procedures in highly complicated cases of adult refractory glaucoma, particularly those associated with severe anterior segment destruction and extensive angle malformations as in anterior staphyloma following trauma, microbial keratitis and neovascular glaucoma (NVG) was found to have a disappointing outcome following conventional glaucoma surgery.[3–6] Over and above, repeated surgical intervention in the form of combined modulated trabeculectomy and cyclodestructive procedures invariably fail in refractory glaucomas.[7–9] Since the usual line of management, both medical and surgical, have proved to be ineffective, application of various glaucoma drainage devices have been tried in the past. High incidence of hypotony with older generations of glaucoma drainage devices deterred ophthalmologists to include them in their armamentarium against cases of refractory glaucoma. The newer generations of glaucoma drainage devices have been found to have significant improvement in terms of design and materials. These changes have encouraged wider acceptance of such devices in refractory glaucomas.[10,15]

In view of the above, an attempt has been made in this study to critically analyze and evaluate the safety and efficacy of Ahmed glaucoma valve (AGV) drainage devices implantation surgery in providing reduction of intra ocular pressure (IOP) in cases of adult refractory glaucomas in the Indian population.

## Materials and Methods

This retrospective study comprised a review of 52 eyes of 32 patients in the age group of 35 to 60 years of age who underwent AGV with or without combined surgical procedures like phacoemulsification with intraocular lens (IOL) implantation, penetrating keratoplasty (PK) or anterior chamber (AC) reconstruction by a single surgeon in a tertiary care setup during the period of January 2003 to January 2007. Ahmed glaucoma valve (FP 7) 184 mm^2^ was used in all cases.[13,15]

Out of these 52 eyes, 46 eyes (88%) had undergone filtering surgery earlier where as the remaining six eyes (12%) had undergone primary AGV implantation following failure of maximal medical therapy.

The common causes of adult refractory glaucoma were found to be late onset juvenile glaucoma in six cases, post-traumatic glaucoma in 12 cases, post- surgical secondary glaucoma (including post PK glaucoma) in eight cases, refractory angle closure glaucoma in 15 cases and refractory open angle glaucoma in 11 cases.

The follow up ranged between 12 months to 48 months (extended up to Jun 2008).

Intraoperative constraints, postoperative control of IOP after one year of surgery, complications and their management were evaluated. The efficacy of IOP control, success as per criteria was defined as IOP of between 9 to 21 mm Hg without medication, qualified success as IOP between 14 to 21 mm Hg with one or more medication and failure as a sustained secondary rise of post op IOP more than 21 mm Hg (once stabilized to an optimal satisfactory level) despite additional support of anti glaucoma medications up to three drugs regimen for more than one month. The outcome was also reviewed and compared with published literature related to refractory glaucoma cases.

**Figure 1:**
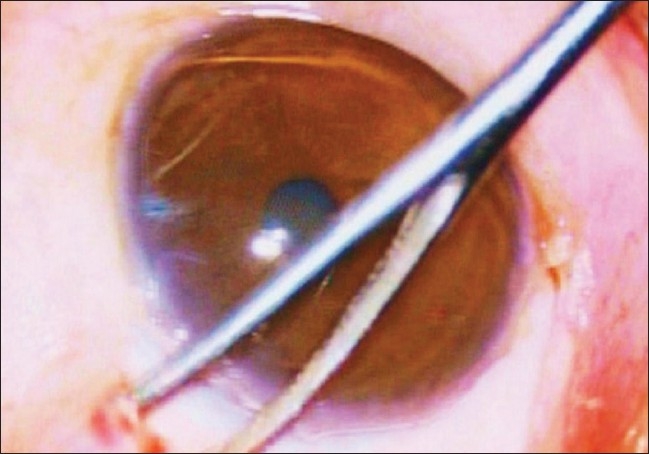
Creation of an episcleral pocket with the help of a vectis

Peribulbar anesthesia was used in all cases. Supero-temporal quadrant was preferred for implant fixation due to the obvious ease of surgical maneuverability.[14,15] After passing a superior rectus bridal suture, a fornix based conjunctival flap was made. A small quantity of 2% lignocaine hydrochloride was injected into the subconjunctival space prior to dissection so as to facilitate separation of the flap. Bipolar cautery was applied to make a good scleral bed for AGV fixation. Wire vectis was used to fashion episcleral pocket [Fig. 1].

**Figure 2:**
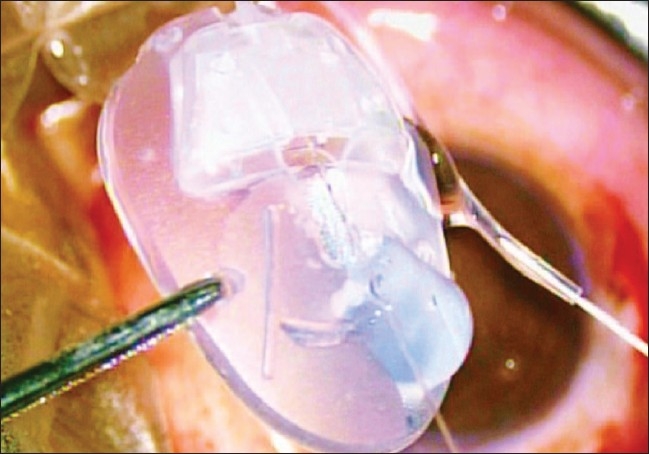
Priming of AG Valve is being carried out with the help of BSS injected through a 30 gauge needle

The AGV was primed with the help of balanced salt solution prior to implantation [Fig. 2].

**Figure 3:**
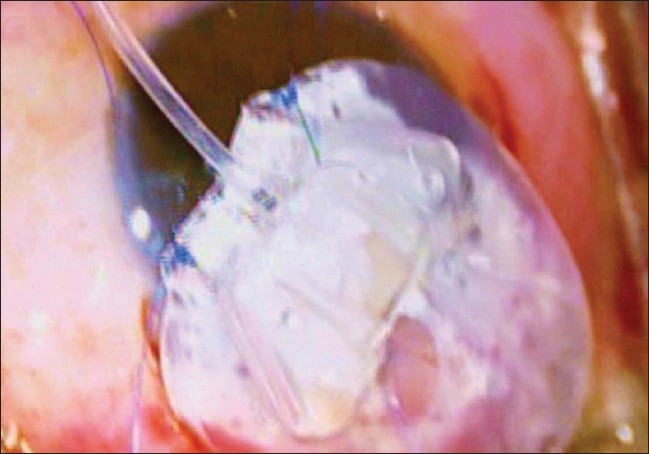
Preplaced 7 ‘0’ Nylon sutures through eyelets for subsequent anchoring of AG Valve over sclera

**Figure 4:**
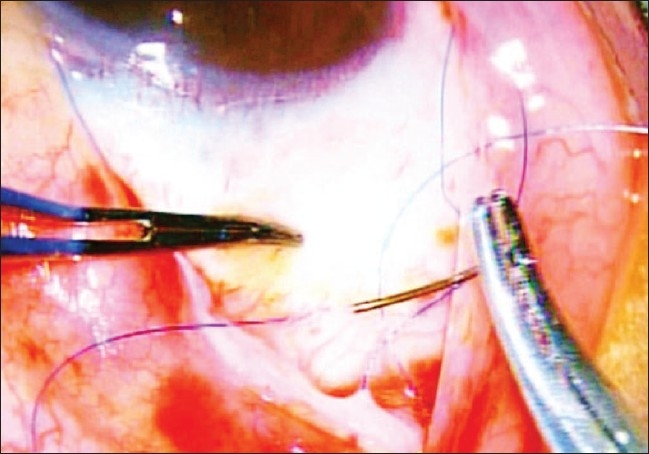
Reverse suturing of 7 ‘0’ Nylon sutures over sclera to secure proper position of AG Valve on the scleral bed

The AGV was anchored 6 to 8 mm behind the limbus with the help of 7/0 prolene suture passed through the eyelets situated in the valve plate [Figs. 3 and 4]. A limbal based partial thickness scleral flap, reaching upto 2/3rds of the scleral thickness, about 3.5 × 3.5 mm square was fashioned to cover the silicone tube of AGV prior to its insertion into the anterior chamber.[13,15] The tube was shortened upto the desired length such that approximately 2- 3 mm protruded into the AC with its bevel facing anteriorly.

**Figure 5:**
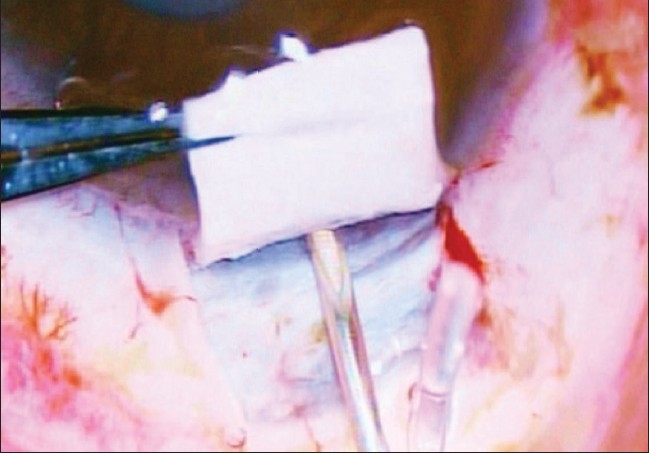
22 Gauge needle track is being constructed for subsequent insertion of AG Valve tube into the anterior chamber. The needle is traversing through the site of trabecular meshwork

**Figure 6:**
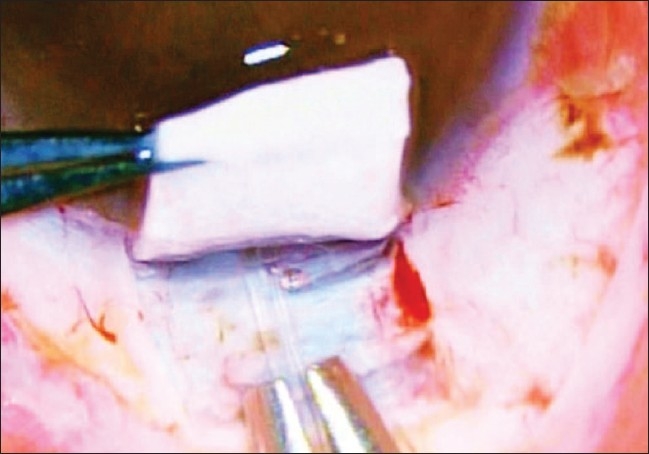
AG Valve tube is introduced into the anterior chamber through the site of trabecular meshwork

**Figure 7:**
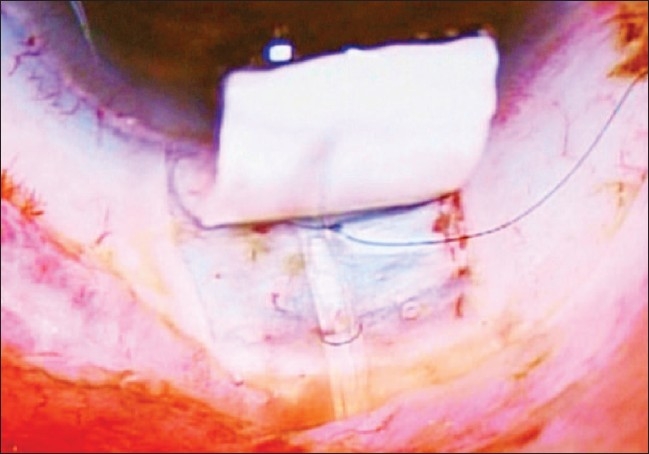
AG Valve tube is anchored with the help of 10'0' Monofilament polyamide suture placed in the form of an elongated box pattern

**Figure 8:**
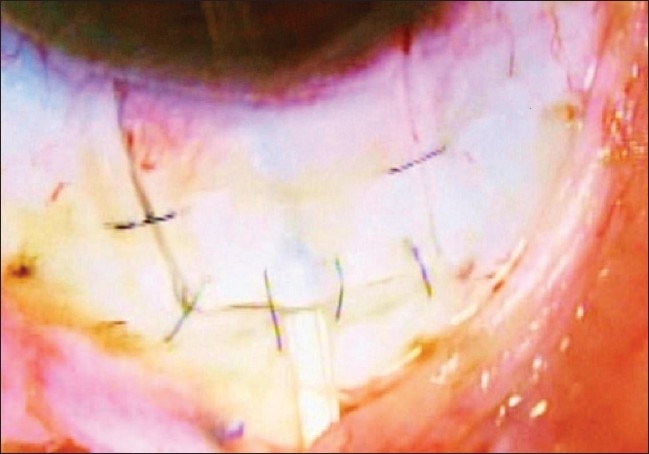
Partial thickness scleral flap is secured over the scleral bed

**Figure 9:**
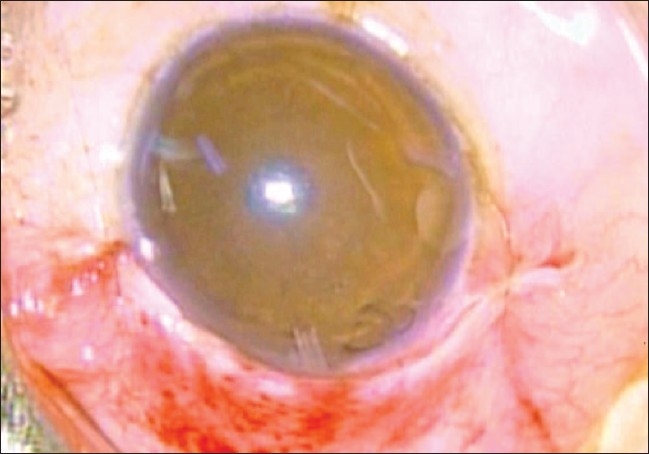
Conjunctival flap is secured with the help of 10'0' Vicryl suture

**Figure 10:**
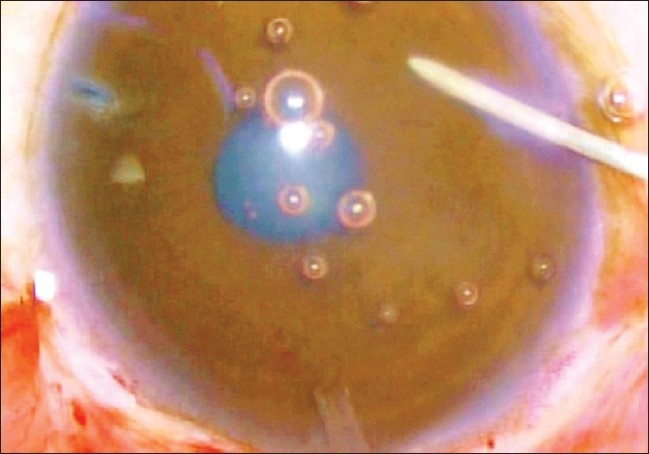
Viscoelastics is being injected into the anterior chamber to maintain adequate IOP during the early post operative phase

The tube of the AGV was introduced into the AC through a sclerostomy made with a 22G needle in the area of the surgical limbus which overlies the trabecular meshwork. The tube was introduced into the AC such that it remained parallel to the iris throughout its course [Figs. 5 and 6]. It was anchored to the sclera beneath the scleral flap by a 10/0 polyamide suture given in a box configuration (surgeon's own modification) [Fig. 7]. Viscoelastics were injected into the AC prior to completion of surgery to ensure a deep anterior chamber and reduce the incidence of hypotony in the early post- operative phase (author's own modification) [Fig. 10]. The partial thickness scleral flap was secured by applying multiple sutures of 10/0 monofilament nylon [Fig. 8]. Conjunctival flap was secured with 10/0 vicryl suture [Fig. 9].

### AGV combined with phacoemulsification:

The surgical technique was partly modified in cases of combined phacoemulsification and AGV surgery. The initial steps short of tube insertion into the AC were performed prior to the commencement of cataract surgery. Phacoemulsification by clear corneal incision using direct chop technique was performed. Single piece hydrophobic acrylic foldable IOL was implanted in all cases. The final step of insertion of AGV tube into AC and subsequent closure of scleral flap was now done as described above.

### Combined with PK:

In cases of concurrent AGV with penetrating keratoplasty, the initial steps short of tube insertion into AC were performed prior to the creation of recipient corneal bed for donor cornea. The tube was inserted into the AC prior to the placement of donor graft corneal button and completion of keratoplasty. Adjuvant procedures like anterior vitrectomy and reconstruction of anterior segment were performed in a usual manner where ever indicated. The remaining steps were identical in all the cases.

Topical dexamethasone and neomycin 0.3% eye drops were given four times daily for 4 weeks and three times in a day for the subsequent two weeks. Moderate cycloplegics like cyclopentolate were administered twice a day for one week followed by once a day for the next week. No antiglaucoma medication was used during the initial phase of hypotony.

**Figure 11:**
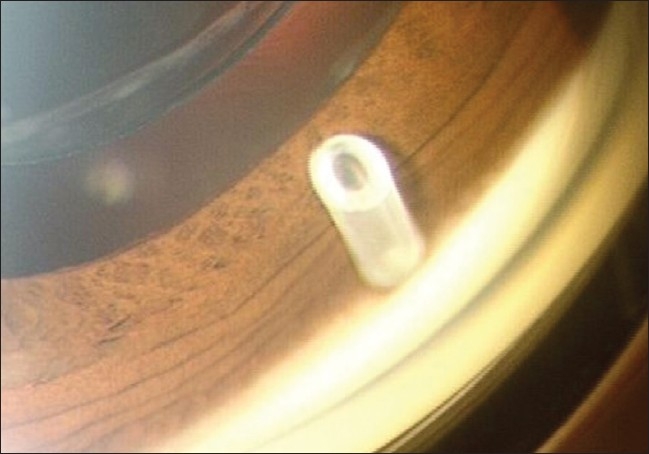
Gonioscopic view of the position of the AG Valve tube into the anterior chamber: Tube is well placed through the trabecular meshwork

Detailed and meticulous postoperative examination was carried out in all cases at regular intervals during follow-up period. The emphasis was given on assessment of visual acuity, extra ocular movements and IOP measurement using non contact tonometer. Detailed slit lamp and fundus examinations were carried out on day 1, 3, once a week for four weeks, monthly for the next six months and thereafter periodically as indicated. Gonioscopic evaluation of AGV tube in AC was carried out at every visit after 8 weeks [Fig. 11]. During follow-up, topical anti- glaucoma medications were added if two consecutive readings of IOP were found to be more than 21 mm Hg. The common medications used to lower postoperative IOP rise in our series were topical beta blockers and topical carbonic anhydrase inhibitors. These drugs were used to maintain adequate IOP control in 4 eyes that had higher IOP despite AGV implantation which is comparable with other studies.

Hypotony was defined as IOP less than 6 mm Hg on any single visit.

## Results

The choice of surgical procedure combined with AGV implantation is depicted in Table 1. We did not notice any significant intraoperative complications except difficulty in inserting AGV tube into the AC in the first attempt in five eyes (10%) and traces of bleeding in three eyes (6%). The mean follow-up period was 20 ± 8 months (range 12 to 42 months). The results were tabulated after one year of follow- up.

**Table 1:** Ahmed glaucoma valve drainage device (AGV) surgery for adult refractory glaucomas: Types of Procedure

Procedure	No. of eyes	%
Phacoemulsification with IOL and AGV implantation	18	35
PK, AGV implant and IOL implantation	13	25
AGV over preexisting IOL implantation	08	15
Secondary IOL with AC reconstruction and AGV implantation.	7	13
AGV implantation only	06	12

In our study, notable post operative complications were secondary hyphema, cystoid macular edema (CME) and Choroidal detachment in three eyes each [Table 2]. Immediate post operative complications seen were exposure of the valve tube following retraction of conjunctival flap in one case and a shallow AC with hypotony in eight eyes.

**Table 2:** Post Operative complications following Ahmed glaucoma valve (AGV) drainage device surgery in cases of adult refractory glaucoma

Post operative complications	No. of eyes	%
Secondary Hyphema	03	6
Choroidal detachment	03	6
Shallow AC with hypotony	08	13
Cystoid macular edema	03	6
Corneal endothelial touch of drainage tube	02	3
Irido- drainage tube adhesions	02	3
Iridocorneal anterior Synechiae	03	5.0
Encapsulated bleb	07	12
Partial exposure of valve plate	01	2

The mean IOP decreased from 36.3 ± 15.7 mm Hg to 19.6 ± 9.2 mm Hg till last follow-up. The number of antiglaucoma medications decreased from a mean of 2.6 ± 1.6 to 1.3 ± 0.7 (*P*<0.001) till last follow-up. Complete success as per criteria was achieved in 46 eyes (88%), where as the remaining six eyes had qualified IOP control with one or two medication. None of the eyes had failure of AGV implantation in terms of IOP control [Table 3]. Forty five (86%) eyes had improvement of visual acuity of more than one line of Snellen's visual acuity chart, while only one eye had a loss of more than one line of Snellen's acuity chart. The remaining cases had no variation in visual acuity.

**Table 3:** Status of intraocular pressure control following Ahmed glaucoma valve (AGV) drainage device surgery in cases of non-responsive refractory glaucomas

Procedure	No. of eyes	IOP Control: Success as per criteria (IOP of between 9 to 21 mm Hg without medication)	Qualified IOP control (IOP between 14 to 21 mm Hg with one or more medication)	Failure= Sustained secondary rise of (Once stabilized to an optimal satisfactory level) post op IOP of >21 mm Hg despite on one or more medication for more than one month
Phacoemulsification with IOL and AGV implantation	18 (35%)	16	02	Nil
AGV implantation only	06 (12%)	06	NIL	Nil
AGV over preexisting IOL implantation	08 (15%)	08	NIL	Nil
PK, AGV implant and IOL implantation	13 (25%)	11	02	Nil
Secondary IOL with				
AC reconstruction and AGV implantation	07 (13%)	05	02	Nil
Total	52	46 eyes (88%)	6 eyes (12%)	Nil

## Discussion

All modern glaucoma drainage devices have the same basic design that consists of a silicone tube leading to a plate or a disc or an encircling element posteriorly beneath the conjunctiva or the Tenon's capsule.[1,2,13–17] The plate or the discs placed posteriorly have a large surface area, which promote formation of a filtering bleb posterior to the equator. The fibrous capsule matures over six months making the bleb thinner. Histologically, the bleb develops microcystic spaces, which serve as channels to shunt the aqueous into orbital tissues. It is this fibrous capsule that offers resistance to aqueous outflow.[1,2,13–15,18,19] Disruption of the conjunctival bleb leads to hypotony. Adequately functioning blebs show slight conjunctival flush and have diffuse edges. Fibrosed blebs appear to be more avascular in appearance and their edges are well defined. They are invariably associated with higher IOP's. In our view, mitomycin C modulation of the bleb surface is of immense value to minimize bleb related complications like fibrosed bleb and failure to maintain IOP control.

We have observed adequate IOP control in all cases of refractory glaucoma. The dependency on anti-glaucoma medications was also decreased significantly.

In our series, formation of an encapsulated bleb postoperatively has been found to be a common observation. Needle puncture was performed in seven (12%) cases of encapsulated bleb with gratifying results.[20] Bleb excision, as mentioned by other workers, was not required in our series.[19,20]

Devices with open tube design are likely to have hypotony in the early post-operative period; whereas the valved devices develop a hypertensive phase from two weeks to six months post-operatively. The persistent hypotensive phase for a prolonged period as reported to be as high as 20–40% in other implant series was not observed in our series.[21] We have successfully prevented early post-operative hypotony with its attendant complications by injecting visco-elastics in the AC at conclusion of surgery.

Peritubular filtration is another cause of severe hypotony after AGV implantation. Improper, ragged and wide sclerostomy track for insertion of AGV tube into the AC is a major cause of concern.[22,23] Leakage from tube insertion site was prevented by making an extremely small entry with the help a 22G needle. To insert the valve tube through such a tight opening was difficult. Visco- dilatation of the needle tract was found to be helpful in passing the tube into the AC.

Creating a scleral flap at the site of tube insertion with use of box anchor suture was found to be beneficial in many ways and possibly there were no incidences of tube exposure in our study as compared to studies where preserved sclera and pericardium were used to cover the tube entry site into AC.[24,25]

The AGV implant has the advantage of having a lower incidence of hypotony without performing a two-staged surgical procedure or any modification in the surgical technique.

Another complication encountered was retraction of the conjunctival flap with barring of implant. This was accompanied by persistent hypotony. Retraction of conjunctiva over the AGV prevents bleb formation. Poor anchoring of AGV to the sclera was noted and movement of the AGV prevented anchorage of the conjunctival flap. We suggest that whenever conjunctival flap retraction occurs, meticulous search for poor anchorage of AGV be carried out. Replacing the displaced valve with a new valve was a better option because growth of fibrous tissue into the AGV prevents smooth opening and closing of the valve plate, thereby rendering it ineffective.

We had observed fine adhesions between the iris and drainage tube in two cases and fine irido-corneal anterior synechiae in three cases. Such adhesions were easily cut with Nd: YAG laser.[25–27]

Failure of opening of the valve plate is another cause of valve failure. Priming and inspection of valve plate and tube prior to its insertion is a crucial step to avoid such preventable complications.[28] Endophthalmitis was not noted in any case in our series.[29]

Das *et al*. conducted a study in 64 patients in north India where results of combined procedure (extra capsular cataract extraction with posterior chamber IOL implantation and AGV implantation) was done. They have reported good IOP control in all cases despite complications. Visual recovery was 20/40 or better in cases who did not have significant glaucomatous cupping pre- operatively.[30] This correlates well with results of our study. However, we did not have any cases of severe post- operative uveitis as reported by them. This difference has arisen from the fact that we did not include any cases of phacomorphic glaucoma in our study.

To conclude, AGV was found to be very effective in the management of refractory glaucomas irrespective of age and etiology, which provides good control of IOP with low incidence of complications.
